# Genetic Pigmentary Disorders: From Molecular Mechanisms to Clinical Manifestations

**DOI:** 10.1111/1346-8138.70024

**Published:** 2025-10-23

**Authors:** Ken Okamura, Tamio Suzuki

**Affiliations:** ^1^ Department of Dermatology Faculty of Medicine, Yamagata University Yamagata Japan; ^2^ Omoriekimae Hifuka Clinic Tokyo Japan

**Keywords:** melanin, melanocytes, oculocutaneous albinism, piebaldism, pigmentation disorders, rASopathies, waardenburg syndrome

## Abstract

Genetic pigmentary disorders represent a diverse group of genetic conditions characterized by alterations in melanin production and transport and melanocyte development, resulting from single‐gene pathological variants. These disorders encompass both hypopigmentary and hyperpigmentary phenotypes, affecting not only skin pigmentation but also ocular, auditory, and systemic manifestations. This review examines the molecular mechanisms underlying major genetic pigmentary disorders, including hypopigmentary (e.g., oculocutaneous albinism, piebaldism, and Waardenburg syndrome) and hyperpigmentary (e.g., dyschromatosis symmetrica hereditaria, dyschromatosis universalis hereditaria, reticulate acropigmentation of Kitamura, and Dowling–Degos disease) disorders. Additionally, we discuss RASopathies, in which pigmentary abnormalities occur alongside multisystem developmental anomalies. Comprehensive understanding of these conditions can provide crucial insights into melanocyte biology and guide future clinical management strategies for affected patients.

## 1 Introduction

Human skin pigmentation is a complex biological process primarily mediated by melanocytes, specialized neural crest‐derived cells residing in the skin epidermis and hair follicles, which synthesize and distribute melanin pigments within melanosomes, specialized lysosome‐related organelles (LROs) that function as the primary sites of melanogenesis [1, 2, 3]. Melanocyte development involves migration from the neural crest under the control of key signaling pathways, including the stem cell factor/c‐KIT, endothelin, Wnt, and α‐melanocyte‐stimulating hormone/melanocortin‐1 receptor pathways, with microphthalmia‐associated transcription factor serving as the master transcriptional regulator [4]. Melanin biosynthetic pathway begins with tyrosine conversion to DOPAquinone by tyrosinase (TYR), leading to eumelanin (brown–black) or pheomelanin (red–yellow) synthesis, followed by melanosome transport and melanin transfer to keratinocytes.

Pathological variants in genes regulating these processes result in genetic pigmentary disorders (GPDs) [5]. These disorders are broadly categorized into hypopigmentary disorders, including oculocutaneous albinism (OCA), piebaldism, and Waardenburg syndrome (WS), and various hyperpigmentary disorders, such as dyschromatosis symmetrica hereditaria (DSH), dyschromatosis universalis (DUH), reticulate acropigmentation of Kitamura (RAK), and Downling–Degos disease (DDD). Additionally, RASopathies represent an important group of disorders caused by germline variants in genes encoding components of the RAS–mitogen‐activated protein kinase (MAPK) signaling pathway, which manifest as diverse pigmentary phenotypes, including café‐au‐lait macules and lentigines, along with characteristic systemic developmental abnormalities. The clinical significance of GPDs extends beyond cosmetic concerns, as many conditions exhibit multisystem manifestations requiring comprehensive medical management [5].

Recent advances in genetic sequencing technologies have significantly enhanced our understanding of the genetic landscape of these disorders, revealing novel disease genes and elucidating complex genotype–phenotype relationships [6, 7, 8]. This review provides a comprehensive overview of the molecular basis, clinical features, and diagnostic approaches for major GPDs and emphasizes the importance of genetic testing for accurate diagnosis, patient management, and genetic counseling.

## 2 Hypopigmentary Disorders

### 2.1 OCA

OCA is an autosomal recessive genetic disorder characterized by the congenital reduction or absence of melanin in the eyes, skin, and hair due to pathogenic variants of the genes involved in melanin biosynthesis. It is broadly classified into nonsyndromic and syndromic subtypes. Nonsyndromic subtypes present with only pigment reduction and ocular symptoms, whereas syndromic subtypes are accompanied by other systemic complications [6]. Albinism can also present as ocular albinism, affecting only the eyes, or as foveal hypoplasia, optic nerve decussation defects, and anterior segment dysgenesis syndrome (FHONDA) [9]. Table 1 shows a list of OCA subtypes and their causative genes identified to date. Nonsyndromic OCA is caused by abnormalities in gene groups directly affecting melanin synthesis, with seven causative genes and one causative locus identified to date. In contrast, syndromic OCA subtypes, including Hermansky–Pudlak syndrome (HPS) and Chédiak–Higashi syndrome (CHS), are caused by abnormalities in genes involved in the biogenesis, trafficking, and function of LROs, including melanosomes. Eleven causative genes for HPS have been identified to date. The causative gene for CHS is *LYST*. In the following sections, we describe each nonsyndromic and syndromic OCA subtype in detail based on the statistical data of Japanese patients.

**TABLE 1:** Causative genes/loci for albinism.

Phenotype (OMIM no.)	Gene (OMIM no.)	
1. Nonsyndromic OCA		
OCA1	*TYR*	(*606933)
OCA1A (#203100)		
OCA1B (#606952)		
OCA2 (#203200)	*OCA2*	(*611409)
OCA3 (#203290)	*TYRP1*	(*115501)
OCA4 (#606574)	*SLC45A2*	(*606202)
OCA5 (%615 312)	4q24	
OCA6 (#113750)	*SLC24A5*	(*609802)
OCA7 (#615179)	*LRMDA*	(*614537)
OCA8 (#619165)	*DCT*	(*191275)
2. Syndromic OCA		
a. Hermansky–Pudlak syndrome (HPS)		
HPS1 (#203300)	*HPS1*	(*604982)
HPS2 (#608233)	*AP3B1*	(*603401)
HPS3 (#614072)	*HPS3*	(*606118)
HPS4 (#614073)	*HPS4*	(*606682)
HPS5 (#614074)	*HPS5*	(*607521)
HPS6 (#614075)	*HPS6*	(*607522)
HPS7 (#614076)	*DTNBP1*	(*607145)
HPS8 (#614077)	*BLOC1S3*	(*609762)
HPS9 (#614171)	*BLOC1S6*	(*604310)
HPS10 (#617050)	*AP3D1*	(*607246)
HPS11 (#619172)	*BLOC1S5*	(*607289)
b. Chediak–Higashi syndrome (#214500)		
	*LYST*	(*606897)
3. Others		
Ocular albinism type 1 (#300500)		
	*GPR143*	(*300808)
FHONDA (#609218)	*SLC38A8*	(*615585)

Abbreviations: FHONDA, foveal hypoplasia, optic nerve decussation defects, and anterior segment dysgenesis; OMIM, Online Mendelian Inheritance in Man.

### 2.2 Nonsyndromic OCA

**FIGURE 1:**
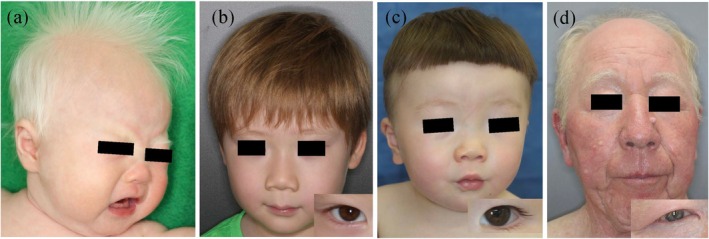
Clinical features of patients with various nonsyndromic oculocutaneous albinism (OCA) subtypes. (a) A 2‐month‐old girl with OCA1A presents with white to blond hair and light skin. She harbors compound heterozygous variants in *TYR*: NM_000372.5:C.929dupC (p.Arg311fs) and c.1292C>T (p.Pro431Leu). (b) A 6‐year‐old boy with OCA1B shows light brown hair and irises with compound heterozygous variants in *TYR*: NM_000372.5:C.230G>A (p.Arg77Gln) and c.1037‐7 T>A. He exhibits no nystagmus but shows mild amblyopia. (c) A 1‐year and 9‐month‐old boy with OCA2 presents with light brown hair and irises. He exhibits photophobia, but not nystagmus. He harbors compound heterozygous variants in *OCA2*: NM_000275.3:C.1182G>A (p.Met394Ile) and c.1441G>A (p.Ala481Thr). (d) A 74‐year‐old man with OCA4 born to consanguineous parents shows blond hair, gray irises, and severe ocular manifestations. He harbors a homozygous variant in *SLC45A2*: NM_016180.5:C.469G>A (p.Asp157Asn).

Nonsyndromic OCA includes eight reported subtypes: OCA1–8. In Japanese patients, OCA4 is the most common (23.1%), followed by OCA1 (19.7%) and OCA2 (10.3%) [10]. Additionally, only three cases of OCA3 and one case of OCA6 have been reported to date [11, 12, 13]. OCA1 is clinically further divided into OCA1A and OCA1B. Patients with OCA1A exhibit characteristic clinical features. Due to loss‐of‐function variants in the causative gene *TYR*, which encodes the rate‐limiting enzyme in melanin synthesis, they completely lack melanin‐synthesizing capacity, which results in white hair and milky white skin, with no pigment enhancement (tanning) upon UV exposure (Figure 1a). Ocular symptoms, such as nystagmus, photophobia, and amblyopia, are also severe, significantly reducing the patient's quality of life. In contrast, OCA1B retains some TYR activity; therefore, degree of pigment reduction is milder and ocular symptoms are often less severe in this subtype than in OCA1A (Figure 1b). Other subtypes also present varying degrees of severity depending on the variant type. Variant NM_000275.3:c.1441G>A (p.Ala481Thr) is present at an exceptionally high frequency in Japanese individuals with OCA2, whereas its allelic frequency is 0.12 in healthy individuals [14]. This variant retains approximately 70% of its function and is associated with the Japanese skin color [15]. Many Japanese patients with OCA2 are compound heterozygotes for the Ala481Thr variant and another pathogenic variant, typically showing mild clinical symptoms (Figure 1c). As mentioned above, OCA4 is the most common subtype among Japanese individuals, likely due to the high frequency of the loss‐of‐function variant NM_016180.5:c.469G>A (p.Asp157Asn) and presence of promoter variant NM_016180.5:c.‐492_489delAATG that retains some function in these individuals [16, 17]. The severity of OCA4 varies depending on the variant type and combination; however, patients with null variants, such as the homozygous Asp157Asn variant, exhibit severe pigment reduction and ocular symptoms (Figure 1d). The causative gene for OCA3 encodes TYRP1, a downstream enzyme in melanin synthesis specifically involved in eumelanin production, which generally results in mild clinical symptoms. In contrast, OCA6 is characterized by mild pigment reduction but severe ocular symptoms [13].

### 2.3 Syndromic OCA

**FIGURE 2:**
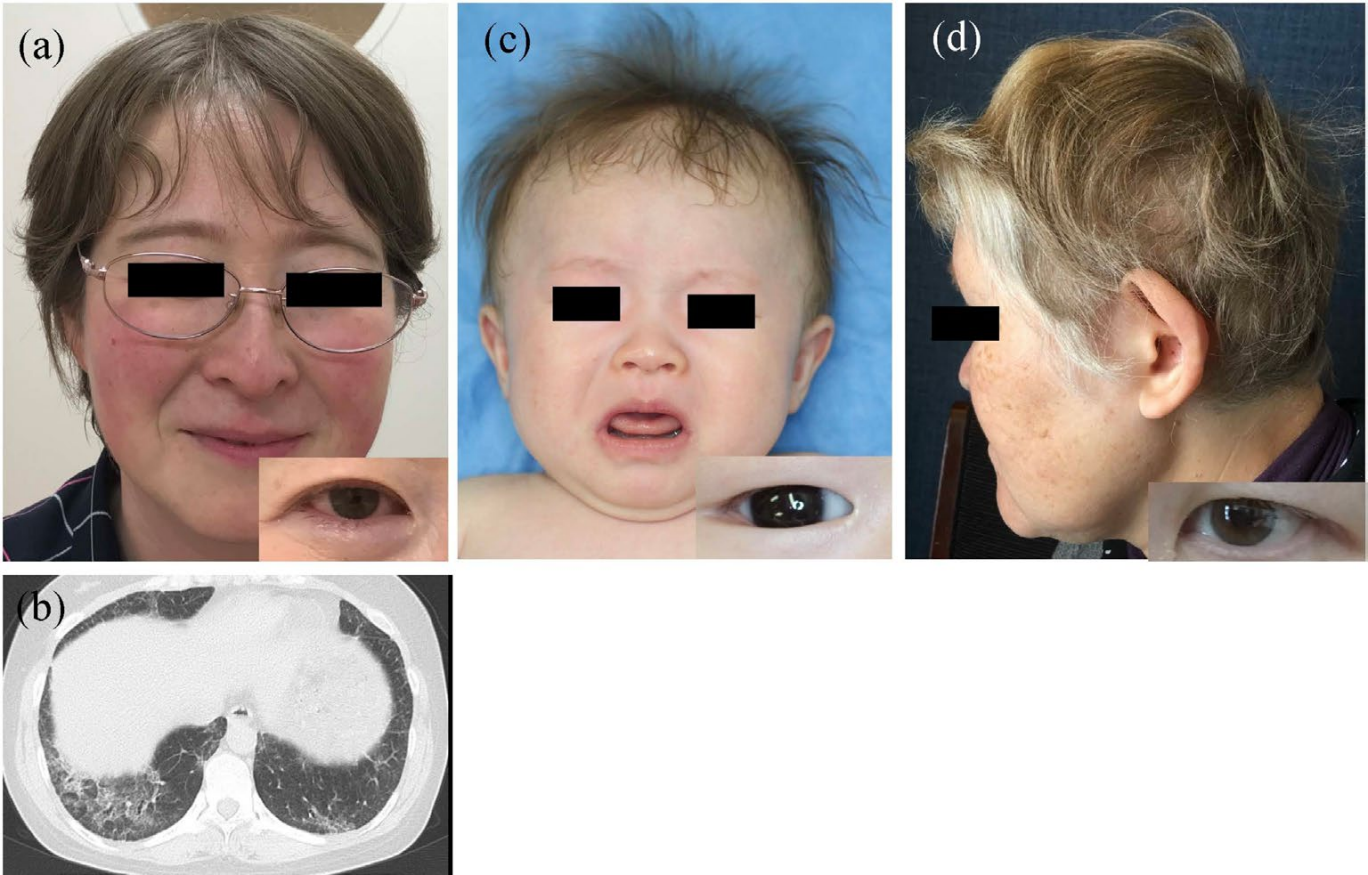
Clinical features of patients with various subtypes of the Hermansky–Pudlak syndrome (HPS). (a and b) A 44‐year‐old woman with HPS1 presents with light brown hair and hazel irises. She exhibits severe eye manifestations, including nystagmus and amblyopia. She harbors compound heterozygous variants in *HPS1*: NM_000195.5:C.1182delT (p.Leu395CysfsTer4) and c.2003 T>C (p.Leu668Pro). Computed tomography revealed pulmonary fibrosis. (c) A 9‐month‐old girl with HPS6 born to nonconsanguineous parents shows mild hypopigmentation and severe ocular manifestations, including nystagmus and amblyopia. She harbors a homozygous variant in *HPS6*: NM_024747.6:C.2038C>T (p.Gln680Ter). (d) A 52‐year‐old woman with HPS9 born to consanguineous parents presents with blond hair and light brown irises. She harbors a homozygous variant in *BLOC1S6*: NM_012388.4:C.285_286dupTC (p.His96LeufsTer22). She also presents with mild leukopenia, thrombocytopenia, and schizophrenia.

HPS is a syndrome in which the membrane transport of proteins necessary for LRO function is impaired. These LROs include melanosomes in melanocytes, platelet‐dense granules in platelets, lamellar bodies in lung pneumocytes, lytic granules in cytotoxic T and natural killer cells, and other specialized organelles. HPS is caused by functional abnormalities in the protein complexes involved in membrane transport, with four protein complexes reported to date: Biogenesis of LRO complex (BLOC)‐1, BLOC‐2, BLOC‐3, and adaptor protein (AP)‐3. Clinical symptoms of HPS result from the impaired function of these protein complexes. Notably, HPS1 accounts for > 10% Japanese OCA cases [10], primarily explained by the founder variant NM_000195.5:c.398 + 5G > A in *HPS1*, which represents approximately half of all pathogenic variants in Japanese patients with HPS1 [6, 18]. HPS1, along with HPS4, constitutes the BLOC‐3 protein, which we refer to here as BLOC‐3 disease. Patients with BLOC‐3 disease present with severe pigment reduction and ocular symptoms and frequently develop fatal pulmonary fibrosis in adulthood (often after middle age; Figure 2a,b). Currently, lung transplantation is the only effective treatment [19]; however, novel therapeutic approaches, including gene therapy strategies, may be developed in the future. BLOC‐2 disease (HPS3, HPS5, and HPS6) is characterized by mild pigment reduction but severe ocular symptoms (Figure 2c) [6]. BLOC‐2 deficiency typically presents without severe systemic complications, with platelet aggregation function often remaining intact [20, 21, 22]. This preservation of function is possibly due to the position of BLOC‐2 downstream from BLOC‐1 in the cellular cargo transport pathway [23]. In contrast, patients consistently develop critical ocular abnormalities, including nystagmus and amblyopia. This clinical pattern suggests that BLOC‐2 plays a disproportionately important function in the retinal pigment epithelium compared to that in neural crest cell‐derived melanocytes. BLOC‐1 disease (HPS7, HPS8, HPS9, and HPS11) is extremely rare worldwide, with only one Japanese case of HPS9 reported to date (Figure 2d) [24]. The severity of OCA varies and is possibly associated with schizophrenia [24, 25]. AP‐3 disease (HPS2 and HPS10, caused by variants in *AP3B1* and *AP3D1*, respectively) is characterized by immunodeficiency in addition to the classic HPS features. Both subtypes can develop interstitial pneumonia; however, HPS10 is distinguished by severe neurological manifestations, including seizures, neurodevelopmental delays, and hearing impairment. This neurological involvement in HPS10 is because *AP3D1* encodes the δ subunit essential for both the ubiquitous and neuronal forms of the AP3 complex. In contrast, AP3*β*3A subunit affected in HPS2 is substituted by AP3*β*3B in the neuron‐specific heterotetramer, allowing neuronal AP3 function to be preserved in patients with HPS2 [26].

CHS develops due to the enlargement of LROs, characterized by giant granules in leukocytes and giant melanosomes in melanocytes, and dysfunction caused by functional abnormalities in the lysosomal trafficking regulator [27]. A distinctive clinical feature differentiating CHS from other OCA subtypes is the coexistence of hypopigmentation and hyperpigmentation following sun exposure, a phenomenon especially noticeable in Asian patients [28, 29]. Additionally, their hair characteristically exhibits a silver‐gray luster and is called “silver‐gray hair.” As it is accompanied by severe complications, such as immunodeficiency, central nervous system symptoms, and hemophagocytic syndrome, early bone marrow transplantation is essential in CHS. Griscelli syndrome (GS) also presents with similar silver‐gray hair due to defective melanosome transport caused by pathological variants of *MYO5A* (GS1), *RAB27A* (GS2), or *MLPH* (GS3). However, unlike classic OCA, in which melanin biosynthesis is impaired, GS affects the intracellular transport of melanosomes, leading some to classify it separately from true albinism [30].

### 2.4 Piebaldism

Piebaldism is an autosomal dominant disorder characterized by partially depigmented patches due to abnormal migration or survival of melanocyte precursor cells and melanoblasts during embryonic development. It is caused by pathogenic variants in *KIT*, which encodes a receptor tyrosine kinase. It is characterized by a triangular white forelock in the central forehead region, present from birth. Depending on the severity, various degrees of depigmented patches and café‐au‐lait macules are observed on the trunk and limbs. No systemic complications are observed. The severity of depigmented patches and café‐au‐lait macules is primarily determined by the location and type of genetic variants. KIT receptor forms dimers that bind to ligands. When pathogenic variants result in abnormal receptors inhibiting dimer formation, this leads to 50% functional loss (haploinsufficiency), resulting in relatively mild symptoms. Conversely, when dimer formation is preserved but signal transduction is impaired, abnormal receptors also inhibit the function of wild‐type receptors (dominant negative effect), causing 75% functional loss and a tendency toward severe manifestations [31]. However, severity can vary even within families carrying the same variant [32], suggesting the influence of other modifier genes or epigenetic factors.

### 2.5 WS

**FIGURE 3:**
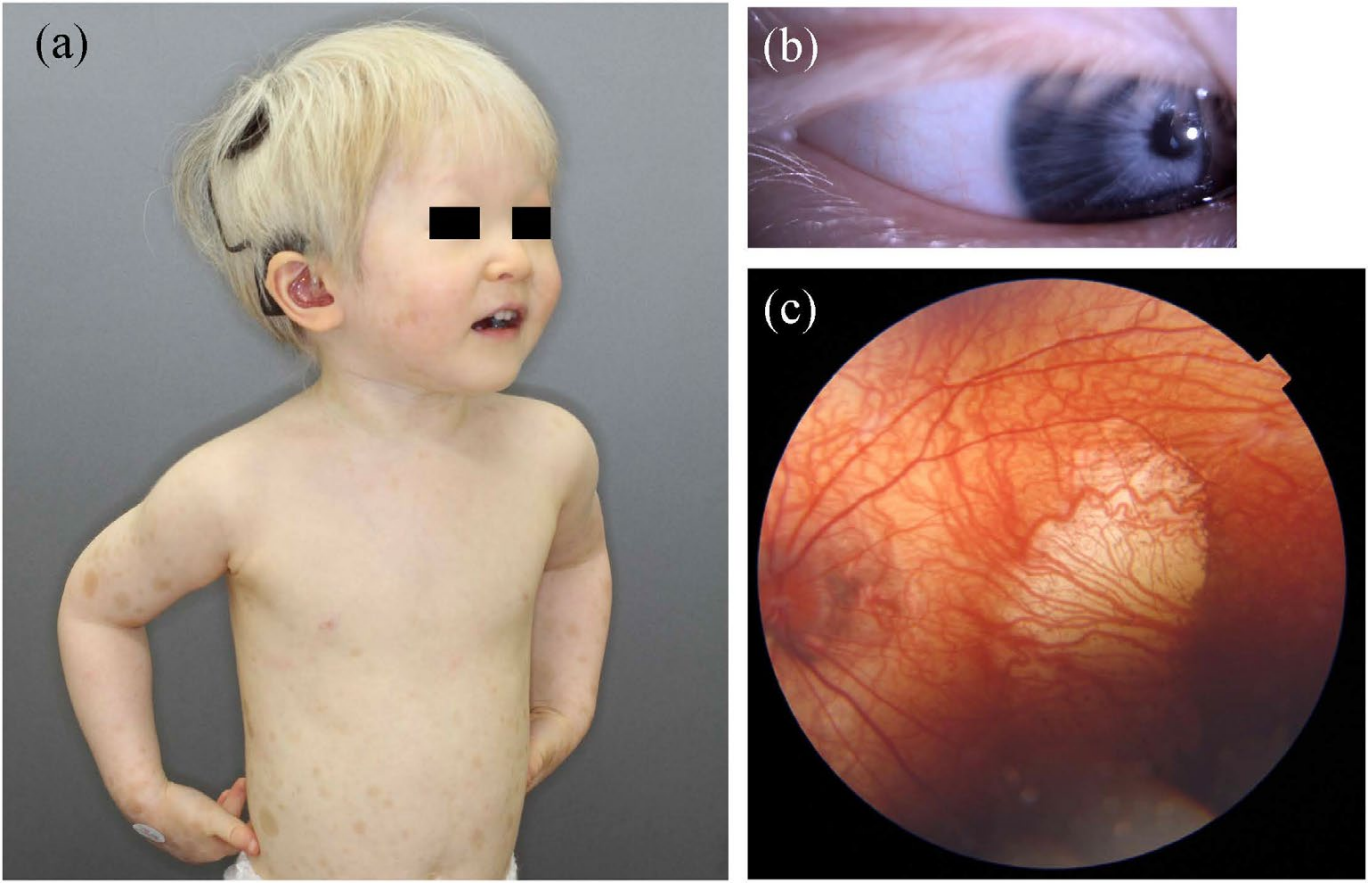
Clinical features of a patient with the Tietz albinism‐deafness syndrome. (a and b) A 3‐year‐old girl presents with blond hair, gray irises, light skin with café‐au‐lait‐like pigmentation, and sensorineural hearing impairment. (c) Fundoscopy revealed hypopigmentation and foveal hypoplasia (left eye). She harbors a dominant negative pathogenic variant in *MITF*: NM_000248:C.637G>C (p.Glu213Gln).

WS affects not only melanocytes but also the differentiation and migration of other neural crest‐derived cells, presenting with other organ symptoms in addition to the skin manifestations of piebaldism described above. It is most commonly autosomal dominant, with a global prevalence of approximately 1 in 42 000 individuals. The three cardinal features of this syndrome are (1) pigmentary abnormalities (piebaldism, white forelock, and iris heterochromia), (2) sensorineural hearing loss, and (3) facial characteristics (dystopia canthorum and broad nasal root). A distinctive feature of WS is that its subtypes are classified based on the clinical symptoms rather than the causative genes. In total, WS has four subtypes (WS1–4), which are further subdivided according to the causative genes (Table 2). Special subtypes include the Peripheral demyelinating neuropathy, Central demyelination, WS, and Hirschsprung disease (PCWH) and Tietz albinism‐deafness syndrome caused by the dominant‐negative variants of *SOX10* and *MITF*, respectively [33, 34]. These represent severe forms of WS4 and WS2, with Tietz albinism‐deafness syndrome characterized by generalized hypopigmentation and profound sensorineural hearing loss (Figure 3) [34].

**TABLE 2:** Classification of Waardenburg syndrome (WS) subtypes.

Clinical type	Clinical features	Genetic subtype/special type	Gene
WS1	All three major features present		*PAX3*
WS2	No facial abnormalities	WS2A	*MITF*
		WS2E	*SOX10*
		WS2F	*KITLG*
		TADS	*MITF*
WS3	Upper limb deformities		*PAX3*
WS4	Associated with Hirschsprung disease	WS4A	*EDNRB*
		WS4B	*EDN3*
		WS4C	*SOX10*
		PCWH	*SOX10*

Abbreviations: PCWH, Peripheral demyelinating neuropathy‐Central dysmyelinating leukodystrophy‐Waardenburg syndrome‐Hirschsprung disease; TADS, Tietz‐albinism deafness syndrome.

## 3 Hyperpigmentary Disorders

### 3.1 DSH

**FIGURE 4:**
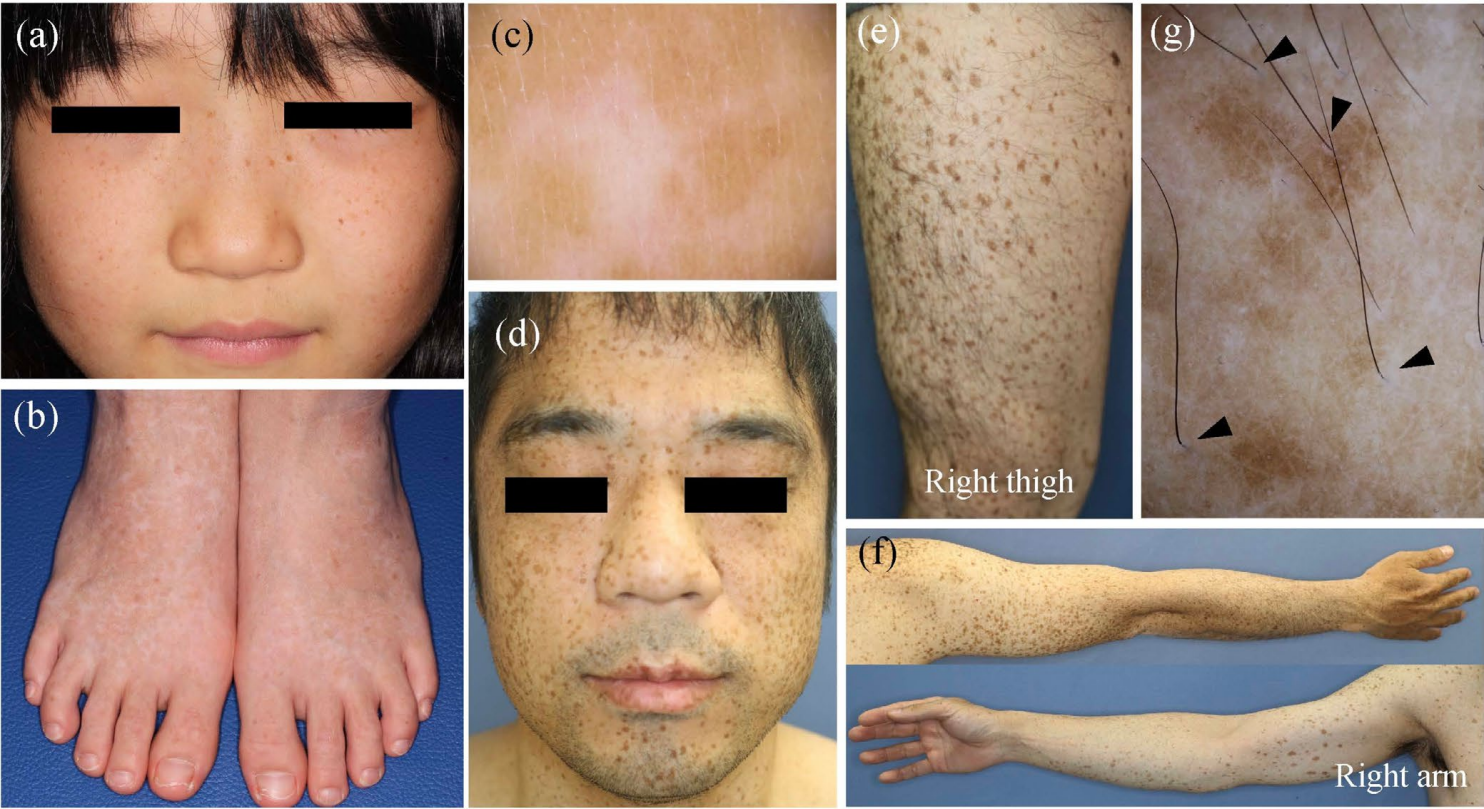
Clinical and dermoscopic features of patients with dyschromatosis symmetrica hereditaria (DSH; a–c) and lentiginous phenotype due to a *SASH1* variant (d–g). (a and b) An 8‐year‐old girl presents with freckle‐like hyperpigmentation on her face and mixed hyper‐ and hypopigmented spots on her dorsal hands and feet. She harbors a heterozygous variant in *ADAR*: NM_001111.5:C.3200 T>G (p.Leu1067Trp). (c) Dermoscopic image of the dorsal foot of the patient with DSH clearly showing mixed hypo‐ and hyperpigmentation. (d–f) A 38‐year‐old man presents with extensive hyperpigmented macules, predominantly on sun‐exposed areas, which are generally larger than the freckle‐like pigmentation observed in patients with DSH. A heterozygous variant was detected in *SASH1*: NM_015278.5:C.1930C>T (p.Arg644Trp). (g) Dermoscopic image of the forearm of the patient with the *SASH1* variant showing irregularly shaped hyperpigmented macules. Interestingly, perifollicular areas show relative hypopigmentation (arrowheads).

DSH is a rare pigmentary genodermatosis inherited via autosomal dominant transmission with high penetrance. This condition is predominantly observed in East Asian countries, particularly Japan, China, and Taiwan, with the first case described by Toyama in 1910 [35]. The clinical presentation of DSH is highly characteristic, featuring intermixed hyper‐ and hypopigmented macules on the dorsal regions of the hands and feet, along with freckle‐like macules on the face (Figure 4a–c). This condition usually manifests in infancy or early childhood as faint hypopigmented macules on the dorsal surfaces of extremities. Subsequently, scattered hyperpigmented macules emerge within the hypopigmented regions. Once established, these lesions persist throughout life without progression or regression, and their color and distribution remain essentially unchanged [35].

Genetic basis of DSH involves pathogenic variants of adenosine deaminase RNA‐specific (*ADAR*), which encodes adenosine deaminase acting on RNA1 (ADAR1) [36]. ADAR1 protein catalyzes the conversion of adenosine to inosine in double‐stranded RNA substrates through a process known as A‐to‐I editing. This protein exists as two isoforms: Interferon‐inducible p150 and constitutively expressed p110 forms. The p150 isoform potentially plays an important role in DSH pathogenesis, as some studies have reported DSH development even with pathogenic variants upstream of the p110 initiation codon, which primarily affects p150 expression while preserving p110 function [37]. Although gene penetrance is reportedly 100% in DSH, clinical features may vary among patients with identical variants, suggesting that environmental factors, such as viral infections or ultraviolet exposure, influence phenotypic expression in this disorder.

Histologically, DSH lesions show a markedly lower number of melanocytes in hypopigmented areas than in normal skin. Electron microscopy has revealed degenerative mitochondrial changes and cytoplasmic vacuole formation in melanocytes, indicative of apoptosis [38]. Its pathogenesis likely involves stress‐induced apoptosis of melanocytes harboring *ADAR* variants, which are potentially triggered by viral infections, leading to the formation of hypopigmented lesions. Subsequently, remnant melanocytes from hair follicle bulge areas possibly migrate to form characteristic hyperpigmented macules.

Notably, *ADAR* is also a causative gene for the Aicardi–Goutières syndrome (AGS), a severe neuroinflammatory disorder characterized by microcephaly, basal ganglia calcification, and intellectual disability [39]. Compound heterozygous *ADAR* variants present with overlapping DSH and AGS phenotypes, where patients exhibit both the characteristic skin manifestations of DSH and neurological symptoms of AGS [40]. This phenotypic spectrum suggests that the degree of ADAR1 functional impairment influences the disease severity and manifestations.

### 3.2 DUH

DUH, first described by Ichikawa and Hiraga in 1933, is characterized by generalized mottled hyperpigmentation and hypopigmentation and distributed throughout the body in a reticulate pattern. This condition typically manifests in infancy or early childhood, presenting with numerous asymptomatic, well‐demarcated, irregular hyperpigmented and hypopigmented macules over the trunk and extremities. DUH is classified into three subtypes: DUH1 (OMIM 127500), DUH2 (OMIM 612715), and DUH3 (OMIM 615402). DUH1 is caused by pathogenic variants in *SASH1*, a tumor suppressor gene. These variants activate the p53/proopiomelanocortin/α‐melanocyte‐stimulating hormone/Gαs/SASH1 signaling cascade [41], which leads to enhanced phosphorylation of extracellular signal‐regulated kinase‐1/2 and CREB, ultimately resulting in hyperpigmentation [42]. Additionally, SASH1 participates in the Gαs/SASH1/IQ motif‐containing GTPase‐activating protein 1/E‐cadherin pathway that regulates melanocyte transepithelial migration [43]. Importantly, *SASH1* variants often present as a distinct clinical entity different from classical DUH. Patients with *SASH1* variants frequently exhibit prominent hyperpigmentation, predominantly on sun‐exposed areas, differing significantly from the balanced hyper‐ and hypopigmented patterns (Figure 4d–g) [44]. Due to this predominantly hyperpigmented presentation, *SASH1*‐related cases are often reported as having a lentiginous phenotype rather than classic DUH [44, 45, 46].

Linkage analysis previously identified 12q21‐q23 as a causative locus for DUH2 [47]. Subsequently, Amyere et al. [48] identified pathogenic variants in *KITLG* on 12q21.34 in patients with familial progressive hyper‐ and hypopigmentation. Considering the similarity of the clinical manifestations of DUH and FPHH, *KITLG* is potentially the causative gene for DUH2.

DUH3 is a classic type of DUH characterized by asymptomatic hyperpigmented and hypopigmented macules showing a generalized distribution over the trunk, limbs, and sometimes the face. In 2013, Zhang et al. identified ATP‐binding cassette subfamily B member 6 gene (*ABCB6*) as the causative gene for this condition [49]. *ABCB6* encodes an ATP‐binding cassette transporter that localizes to the lysosomal and early melanosomal membranes. Two major pathogenic mechanisms of DUH have been identified to date. First, pathogenic variants of *ABCB6* cause altered subcellular localization, with mutant proteins being retained in the Golgi apparatus, rather than being properly trafficked to melanosomes and dendrites [49]. This disrupts melanosome transport and distribution to keratinocytes. Second, ABCB6 variants impair the early steps of melanogenesis by disrupting premelanosome protein amyloid fibril formation in stage II melanosomes [50]. Premelanosome protein fibrils serve as the structural scaffold for eumelanin deposition, and their disruption leads to aberrant protein aggregation and defective melanosome maturation. These defects possibly contribute to abnormal melanosome function, potentially affecting melanin synthesis, storage, and distribution, which explains the characteristic mixed hyperpigmented and hypopigmented macules observed in patients with DUH. Recently, variants in the period circadian regulator 3 gene (*PER3*), particularly the *PER3* rs772027021 single‐nucleotide variant, have been reported to be associated with the DUH phenotype [51]. This variant may represent a novel DUH subtype, potentially designated as DUH4, characterized by milder pigmentation abnormalities [52].

### 3.3 RAK

**FIGURE 5:**
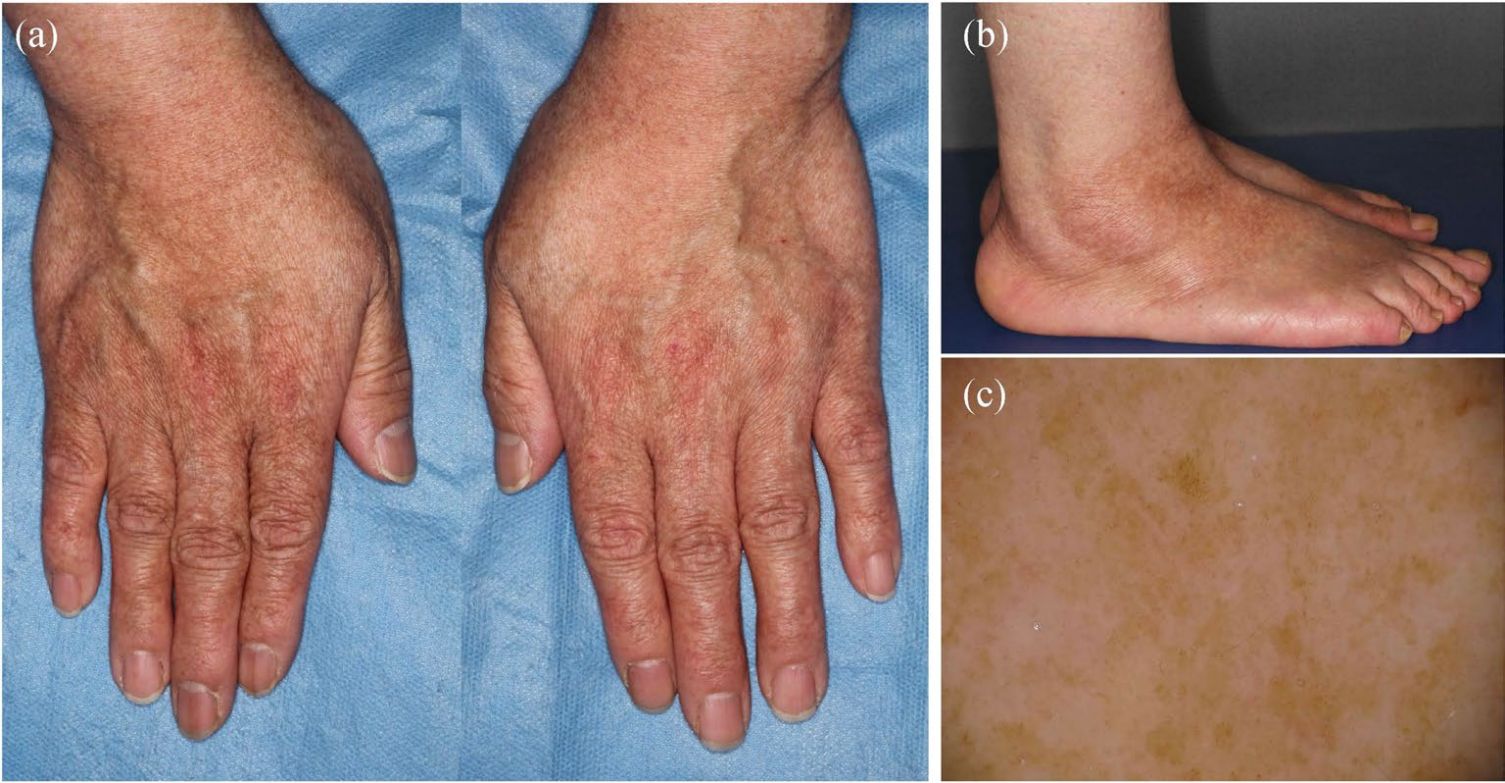
Clinical features of a patient with reticulate acropigmentation of Kitamura (RAK). (a and b) Reticulate hyperpigmentation on the dorsum of the extremities. (c) Dermoscopic image of hyperpigmented spots showing irregularly shaped hyperpigmentation, without any hypopigmentation. A heterozygous variant was detected in *ADAM10*:NM_001110.4: C.1931G>C (p.Arg644Pro).

RAK is a rare autosomal dominant pigmentary disorder caused by variants in *ADAM10*, which encodes a zinc metalloprotease involved in ectodomain shedding of various membrane proteins [53]. First described by Kitamura in 1943, RAK typically manifests in the first or second decade of life with sharply demarcated, reticulate, and slightly depressed brown macules primarily affecting the dorsa of hands and feet (Figure 5) [54]. The macules gradually darken and extend proximally, progressing until middle age before disappearing in the 70s. Additional features include breaks in the epidermal ridges of palms and fingers, palmoplantar pits, and occasional plantar keratoderma and partial alopecia [55].

Histologically, RAK exhibits epidermal thinning with elongated and thin rete ridges, showing pigmentation at their pointed ends. The stratum corneum exhibits slight hyperkeratosis without parakeratosis, whereas the superficial dermis shows minimal inflammatory infiltration without pigmentary incontinence. Epidermal melanocyte numbers are increased in this disorder, with electron microscopy showing aggregated melanosomes in melanocytes and an increased number of melanosome complexes in keratinocytes [54].

### 3.4 DDD

DDD is an autosomal dominant genodermatosis caused by variants of multiple genes, including *KRT5*, *POFUT1*, *POGLUT1*, or *PSENEN* [56]. The condition presents with reticulate brown macules similar to RAK but demonstrates distinct clinical and temporal characteristics. DDD typically develops around or after the age of 20 years, primarily affecting the flexural areas and major skin folds, rather than the acral regions. In contrast to the uniform light brown macules of RAK, the pigmented lesions appear brown to black in this condition. The key distinguishing features of DDD include comedo‐like follicular papules, genital region involvement, and characteristic dyschromatosis (mixed small hyper‐ and hypopigmented macules in the affected areas). Patients often experience pruritus in skin lesions, which is atypical of RAK. Histologically, DDD shows acanthosis with tight digitiform rete ridges and notable pigmentary incontinence in the dermis, which is in sharp contrast to the epidermal thinning pattern observed in RAK [57].

Both RAK and DDD disrupt the Notch signaling pathway, which plays an important role in melanocyte homeostasis. ADAM10 functions in the ectodomain shedding of Notch proteins as substrates in the skin, whereas POFUT1 and POGLUT1 are essential components involved in the post‐translational modification of Notch proteins via fucosylation and glucosylation, respectively [55]. The gene product of *PSENEN* also participates in the Notch signaling pathway and is associated with DDD, particularly in cases with concurrent hidradenitis suppurativa [58, 59]. Some case reports have documented patients with DDD with hidradenitis suppurativa harboring pathogenic variants in *NCSTN* or *KRT14* [60, 61]. Impaired Notch signaling disrupts melanocyte homeostasis; however, the precise mechanisms linking Notch pathway dysfunction to the characteristic hyperpigmentation patterns in this condition remain unclear, warranting further research.

### 3.5 RASopathies and Pigmentary Manifestations

RASopathies are developmental disorders affecting approximately 1 in 1000 individuals that are caused by pathogenic variants in genes encoding components of the RAS–MAPK signaling pathway, which plays a crucial role in melanocyte biology [62, 63]. The RAS pathway regulates essential cellular processes, including growth, differentiation, apoptosis, migration, and senescence. Pathogenic variants of RAS‐related genes result in the dysregulation of these signaling cascades, leading to the characteristic developmental abnormalities and pigmentary changes observed in these conditions. Café‐au‐lait macules are the most common pigmentary abnormalities associated with RASopathies. In neurofibromatosis type 1, loss of neurofibromin disrupts the RAS–GTPase‐activating protein function, resulting in constitutive activation of the RAS–MAPK pathway in melanocytes. Similar café‐au‐lait macules also occur in Legius syndrome, which is caused by pathogenic variants in *SPRED1*, reflecting its functional convergence with RAS–MAPK signaling [64]. In contrast, McCune–Albright syndrome is caused by the postzygotic activating variants of *GNAS*, resulting in mosaicism that manifests as large segmental café‐au‐lait macules with jagged “coast of Maine” borders. These lesions follow Blaschko's lines, reflecting the developmental lineage of the affected melanocyte precursors. These pigmentary changes often represent the earliest clinical signs of the McCune–Albright syndrome [65].

**FIGURE 6:**
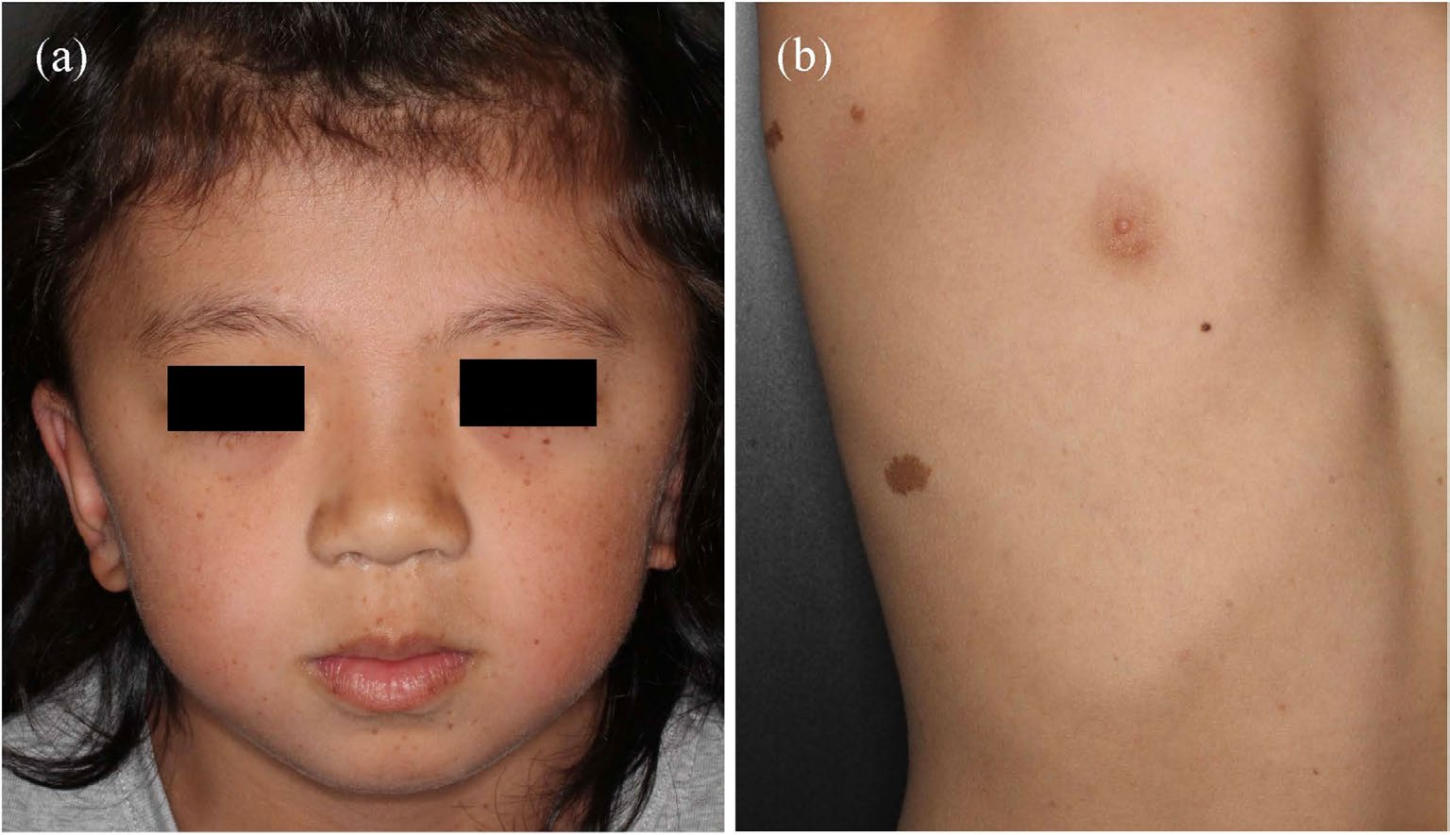
Clinical features of a patient with Noonan syndrome with multiple lentigines. (a) A 6‐year‐old girl presents with multiple lentigines on her face, along with ocular hypertelorism, broad nasal root, and low‐set ears. (b) Café‐au‐lait macules are observed on the patient's body. She harbors a heterozygous variant in *PTPN11*: NM_002834.5:C.1403C>T (p.Thr468Met).

Noonan syndrome and related conditions, including cardiofaciocutaneous syndrome and Costello syndrome, present with various pigmentary abnormalities, including café‐au‐lait macules, lentigines, and distinctive hair anomalies. Pathogenic variants in *PTPN11* are associated with the Noonan syndrome with multiple lentigines, formerly known as LEOPARD syndrome, which is an acronym for Lentigines, Electrocardiographic abnormalities, Ocular hypertelorism, Pulmonary stenosis, Abnormal genitalia, Retardation of growth, and Deafness [64]. Multiple facial lentigines, along with characteristic facial dysmorphism, represent the key diagnostic features of this condition (Figure 6). The therapeutic implications of understanding these mechanisms have become increasingly apparent with the success of MEK inhibitors, such as selumetinib, in treating RASopathy complications [66], directly demonstrating the ways in which molecular insights translate into clinical advances and enhance our understanding of pigmentation regulation via developmental signaling pathways.

## 4 Future Directions and Conclusion

Advances in sequencing technologies have facilitated the identification of novel genes associated with GPDs, enhancing our understanding of the underlying molecular mechanisms and normal pigmentation biology. This increasing genetic knowledge allows the development of precision medicine approaches via improved genotype–phenotype correlations and personalized management strategies based on individual genetic profiles.

GPDs represent a diverse group of genetic conditions that provide important insights into melanocyte biology and pigmentation regulation. Their clinical management requires multidisciplinary approaches addressing not only pigmentary manifestations but also associated complications, such as visual impairment, hearing loss, and cancer predisposition. Early diagnosis and appropriate supportive care remain fundamental for improving the quality of life of the affected individuals. Continued research into these genetic conditions is essential not only for improving patient outcomes but also for advancing our understanding of fundamental cellular mechanisms that extend well beyond melanocyte function, with implications for broader biological processes and therapeutic development.

## Ethics Statement

Written informed consent was obtained from all patients or their legal guardians for publication of clinical photographs presented in this review (Figures 1, 2, 3, 4, 5, 6), under protocols approved by the Ethics Committee of the Faculty of Medicine, Yamagata University. All images were modified to protect patient privacy.

## Conflicts of Interest

Tamio Suzuki is an editorial board member of the journal of Dermatology and a co‐author of this article. To minimize bias, he was excluded from all editorial decision‐making related to the acceptance of this article for publication.

## Data Availability

The data that support the findings of this study are available from the corresponding author upon reasonable request.

## References

[1] Y. Yamaguchi and V. J. Hearing , “Physiological Factors That Regulate Skin Pigmentation,” BioFactors 35 (2009): 193–199. 19449448 10.1002/biof.29 PMC2793097

[2] G. E. Costin and V. J. Hearing , “Human Skin Pigmentation: Melanocytes Modulate Skin Color in Response to Stress,” FASEB Journal 21 (2007): 976–994. 17242160 10.1096/fj.06-6649rev

[3] G. Raposo and M. S. Marks , “Melanosomes–Dark Organelles Enlighten Endosomal Membrane Transport,” Nature Reviews Molecular Cell Biology 8 (2007): 786–797. 17878918 10.1038/nrm2258 PMC2786984

[4] A. Kawakami and D. E. Fisher , “The Master Role of Microphthalmia‐Associated Transcription Factor in Melanocyte and Melanoma Biology,” Laboratory Investigation 97 (2017): 649–656. 28263292 10.1038/labinvest.2017.9

[5] Y. Tomita and T. Suzuki , “Genetics of Pigmentary Disorders,” American Journal of Medical Genetics. Part C, Seminars in Medical Genetics 131C (2004): 75–81. 15452859 10.1002/ajmg.c.30036

[6] K. Okamura and T. Suzuki , “Current Landscape of Oculocutaneous Albinism in Japan,” Pigment Cell & Melanoma Research 34 (2021): 190–203. 32969595 10.1111/pcmr.12927

[7] A. B. Murthy , V. Palaniappan , K. Karthikeyan , and V. Anbarasan , “Dyschromatosis Universalis Hereditaria,” International Journal of Dermatology 62 (2023): 1218–1227. 37634201 10.1111/ijd.16817

[8] L. Mohapatra , K. Sardana , M. Panda , and R. Mahajan , “An Algorithmic Approach Towards Diagnosis of Patients With Hereditary Reticulate Pigmentary Disorders: A Narrative Review,” Clinical and Experimental Dermatology 50 (2024): 12–20. 39139099 10.1093/ced/llae322

[9] A. Fernández , M. Hayashi , G. Garrido , et al., “Genetics of Non‐Syndromic and Syndromic Oculocutaneous Albinism in Human and Mouse,” Pigment Cell & Melanoma Research 34 (2021): 786–799. 33960688 10.1111/pcmr.12982

[10] K. Okamura , T. Saito , N. Oiso , et al., “Updated Analysis of Albinism in Japan: 290 Families With Novel Pathological Variants,” Pigment Cell & Melanoma Research, Under Review. 10.1111/pcmr.70066 41292147

[11] M. Yamada , K. Sakai , M. Hayashi , et al., “Oculocutaneous Albinism Type 3: A Japanese Girl With Novel Mutations in TYRP1 Gene,” Journal of Dermatological Science 64 (2011): 217–222. 21996312 10.1016/j.jdermsci.2011.09.005

[12] K. Okamura , J. Yoshizawa , Y. Abe , et al., “Oculocutaneous Albinism (OCA) in Japanese Patients: Five Novel Mutations,” Journal of Dermatological Science 74 (2014): 173–174. 24461674 10.1016/j.jdermsci.2013.12.011

[13] T. Saito , K. Okamura , R. Kosaki , et al., “Impact of a SLC24A5 Variant on the Retinal Pigment Epithelium of a Japanese Patient With Oculocutaneous Albinism Type 6,” Pigment Cell & Melanoma Research 35 (2022): 212–219. 34870899 10.1111/pcmr.13024

[14] T. Suzuki , Y. Miyamura , and Y. Tomita , “High Frequency of the Ala481Thr Mutation of the P Gene in the Japanese Population,” American Journal of Medical Genetics. Part A 118a (2003): 402–403. 12687678 10.1002/ajmg.a.20044

[15] Y. Abe , G. Tamiya , T. Nakamura , Y. Hozumi , and T. Suzuki , “Association of Melanogenesis Genes With Skin Color Variation Among Japanese Females,” Journal of Dermatological Science 69 (2013): 167–172. 23165166 10.1016/j.jdermsci.2012.10.016

[16] K. Inagaki , T. Suzuki , S. Ito , et al., “OCA4: Evidence for a Founder Effect for the p.D157N Mutation of the MATP Gene in Japanese and Korean,” Pigment Cell Research 18 (2005): 385–388. 16162179 10.1111/j.1600-0749.2005.00261.x

[17] K. Okamura , M. Hayashi , O. Nakajima , et al., “A 4‐Bp Deletion Promoter Variant (rs984225803) is Associated With Mild OCA4 Among Japanese Patients,” Pigment Cell & Melanoma Research 32 (2019): 79–84. 30019506 10.1111/pcmr.12727

[18] S. Ito , T. Suzuki , K. Inagaki , et al., “High Frequency of Hermansky‐Pudlak Syndrome Type 1 (HPS1) Among Japanese Albinism Patients and Functional Analysis of HPS1 Mutant Protein,” Journal of Investigative Dermatology 125 (2005): 715–720. 16185271 10.1111/j.0022-202X.2005.23884.x

[19] P. Velázquez‐Díaz , E. Nakajima , P. Sorkhdini , et al., “Hermansky‐Pudlak Syndrome and Lung Disease: Pathogenesis and Therapeutics,” Frontiers in Pharmacology 12 (2021): 644671. 33841163 10.3389/fphar.2021.644671 PMC8028140

[20] V. Michaud , E. Lasseaux , C. Plaisant , et al., “Clinico‐Molecular Analysis of Eleven Patients With Hermansky‐Pudlak Type 5 Syndrome, a Mild Form of HPS,” Pigment Cell & Melanoma Research 30 (2017): 563–570. 28640947 10.1111/pcmr.12608

[21] T. Saito , K. Okamura , Y. Funasaka , Y. Abe , and T. Suzuki , “Identification of Two Novel Mutations in a Japanese Patient With Hermansky‐Pudlak Syndrome Type 5,” Journal of Dermatology 47 (2020): e392‐e3. 32881069 10.1111/1346-8138.15560

[22] K. Okamura , M. Hayashi , Y. Abe , et al., “NGS‐Based Targeted Resequencing Identified Rare Subtypes of Albinism: Providing Accurate Molecular Diagnosis for Japanese Patients With Albinism,” Pigment Cell & Melanoma Research 32 (2019): 848–853. 31141302 10.1111/pcmr.12800

[23] M. S. Marks and H. F. Heijnen , Raposo G “Lysosome‐Related Organelles: Unusual Compartments Become Mainstream,” Current Opinion in Cell Biology 25 (2013): 495–505. 23726022 10.1016/j.ceb.2013.04.008 PMC3729921

[24] K. Okamura , Y. Abe , Y. Araki , et al., “Characterization of Melanosomes and Melanin in Japanese Patients With Hermansky‐Pudlak Syndrome Types 1, 4, 6, and 9,” Pigment Cell & Melanoma Research 31 (2018): 267–276. 29054114 10.1111/pcmr.12662

[25] Y. Q. Feng , Z. Y. Zhou , X. He , et al., “Dysbindin Deficiency in Sandy Mice Causes Reduction of Snapin and Displays Behaviors Related to Schizophrenia,” Schizophrenia Research 106 (2008): 218–228. 18774265 10.1016/j.schres.2008.07.018

[26] S. Ammann , A. Schulz , I. Krageloh‐Mann , et al., “Mutations in AP3D1 Associated With Immunodeficiency and Seizures Define a New Type of Hermansky‐Pudlak Syndrome,” Blood 127 (2016): 997–1006. 26744459 10.1182/blood-2015-09-671636 PMC7611501

[27] K. Fukai , J. Oh , M. A. Karim , et al., “Homozygosity Mapping of the Gene for Chediak‐Higashi Syndrome to Chromosome 1q42‐q44 in a Segment of Conserved Synteny That Includes the Mouse Beige Locus (Bg),” American Journal of Human Genetics 59 (1996): 620–624. 8751863 PMC1914913

[28] K. Fukuchi , K. Tatsuno , K. Sakaguchi , et al., “Novel Gene Mutations in Chédiak‐Higashi Syndrome With Hyperpigmentation,” Journal of Dermatology 46 (2019): e416‐e8. 31245861 10.1111/1346-8138.14987

[29] K. Fukai , M. Ishii , A. Kadoya , M. Chanoki , and T. Hamada , “Chédiak‐Higashi Syndrome: Report of a Case and Review of the Japanese Literature,” Journal of Dermatology 20 (1993): 231–237. 8315113 10.1111/j.1346-8138.1993.tb03867.x

[30] L. Montoliu and M. S. Marks , “A New Type of Syndromic Albinism Associated With Mutations in AP3D1,” Pigment Cell & Melanoma Research 30 (2017): 5–7. 27900855 10.1111/pcmr.12543 PMC5555751

[31] N. Oiso , K. Fukai , A. Kawada , and T. Suzuki , “Piebaldism,” Journal of Dermatology 40 (2013): 330–335. 22670867 10.1111/j.1346-8138.2012.01583.x

[32] T. Narita , N. Oiso , K. Fukai , et al., “Two Children With a Mild or Moderate Piebaldism Phenotype and a Father Without Leukoderma in a Family With the Same Recurrent Missense Mutation in the Kinase Domain of KIT,” European Journal of Dermatology 21 (2011): 446–447. 21680281 10.1684/ejd.2011.1350

[33] K. Inoue , M. Khajavi , T. Ohyama , et al., “Molecular Mechanism for Distinct Neurological Phenotypes Conveyed by Allelic Truncating Mutations,” Nature Genetics 36 (2004): 361–369. 15004559 10.1038/ng1322

[34] K. Yamamoto , K. Okamura , K. Wakamatsu , et al., “Genetic Insights Into Tietz Albinism‐Deafness Syndrome: A New Dominant‐Negative Mutation in MITF,” Pigment Cell & Melanoma Research 37 (2024): 430–437. 38439523 10.1111/pcmr.13166

[35] M. Hayashi and T. Suzuki , “Dyschromatosis Symmetrica Hereditaria,” Journal of Dermatology 40 (2013): 336–343. 22974014 10.1111/j.1346-8138.2012.01661.x

[36] Y. Miyamura , T. Suzuki , M. Kono , et al., “Mutations of the RNA‐Specific Adenosine Deaminase Gene (DSRAD) are Involved in Dyschromatosis Symmetrica Hereditaria,” American Journal of Human Genetics 73 (2003): 693–699. 12916015 10.1086/378209 PMC1180697

[37] N. Suzuki , T. Suzuki , K. Inagaki , et al., “Ten Novel Mutations of the ADAR1 Gene in Japanese Patients With Dyschromatosis Symmetrica Hereditaria,” Journal of Investigative Dermatology 127 (2007): 309–311. 16917490 10.1038/sj.jid.5700528

[38] T. Kondo , T. Suzuki , Y. Mitsuhashi , et al., “Six Novel Mutations of the ADAR1 Gene in Patients With Dyschromatosis Symmetrica Hereditaria: Histological Observation and Comparison of Genotypes and Clinical Phenotypes,” Journal of Dermatology 35 (2008): 395–406. 18705826 10.1111/j.1346-8138.2008.00493.x

[39] G. I. Rice , P. R. Kasher , G. M. Forte , et al., “Mutations in ADAR1 Cause Aicardi‐Goutieres Syndrome Associated With a Type I Interferon Signature,” Nature Genetics 44 (2012): 1243–1248. 23001123 10.1038/ng.2414 PMC4154508

[40] M. Kono , F. Matsumoto , Y. Suzuki , et al., “Dyschromatosis Symmetrica Hereditaria and Aicardi‐Goutieres Syndrome 6 Are Phenotypic Variants Caused by ADAR1 Mutations,” Journal of Investigative Dermatology 136 (2016): 875–878. 26802932 10.1016/j.jid.2015.12.034

[41] D. Zhou , Z. Wei , Z. Kuang , et al., “A Novel P53/POMC/Gαs/SASH1 Autoregulatory Feedback Loop Activates Mutated SASH1 to Cause Pathologic Hyperpigmentation,” Journal of Cellular and Molecular Medicine 21 (2017): 802–815. 27885802 10.1111/jcmm.13022 PMC5345616

[42] D. Zhou , Z. Kuang , X. Zeng , et al., “p53 Regulates ERK1/2/CREB Cascade via a Novel SASH1/MAP2K2 Crosstalk to Induce Hyperpigmentation,” Journal of Cellular and Molecular Medicine 21 (2017): 2465–2480. 28382689 10.1111/jcmm.13168 PMC5618682

[43] D. Zhou , Z. Wei , S. Deng , et al., “SASH1 Regulates Melanocyte Transepithelial Migration Through a Novel Galphas‐SASH1‐IQGAP1‐E‐Cadherin Dependent Pathway,” Cellular Signalling 25 (2013): 1526–1538. 23333244 10.1016/j.cellsig.2012.12.025

[44] Y. Araki , K. Okamura , T. Saito , et al., “Five Novel Mutations in SASH1 Contribute to Lentiginous Phenotypes in Japanese Families,” Pigment Cell & Melanoma Research 34 (2021): 174–178. 32981204 10.1111/pcmr.12930

[45] Y. G. Shellman , K. A. Lambert , A. Brauweiler , et al., “SASH1 Is Involved in an Autosomal Dominant Lentiginous Phenotype,” Journal of Investigative Dermatology 135 (2015): 3192–3194. 26203640 10.1038/jid.2015.292 PMC4648645

[46] J. Zhang , R. Cheng , J. Liang , C. Ni , M. Li , and Z. Yao , “Lentiginous Phenotypes Caused by Diverse Pathogenic Genes (SASH1 and PTPN11): Clinical and Molecular Discrimination,” Clinical Genetics 90 (2016): 372–377. 27659786 10.1111/cge.12728

[47] M. Stuhrmann , H. C. Hennies , I. A. Bukhari , et al., “Dyschromatosis Universalis Hereditaria: Evidence for Autosomal Recessive Inheritance and Identification of a New Locus on Chromosome 12q21‐q23,” Clinical Genetics 73 (2008): 566–572. 18462451 10.1111/j.1399-0004.2008.01000.x

[48] M. Amyere , T. Vogt , J. Hoo , et al., “KITLG Mutations Cause Familial Progressive Hyper‐ and Hypopigmentation,” Journal of Investigative Dermatology 131 (2011): 1234–1239. 21368769 10.1038/jid.2011.29

[49] C. Zhang , D. Li , J. Zhang , et al., “Mutations in ABCB6 Cause Dyschromatosis Universalis Hereditaria,” Journal of Investigative Dermatology 133 (2013): 2221–2228. 23519333 10.1038/jid.2013.145

[50] P. Bergam , J. M. Reisecker , Z. Rakvacs , et al., “ABCB6 Resides in Melanosomes and Regulates Early Steps of Melanogenesis Required for PMEL Amyloid Matrix Formation,” Journal of Molecular Biology 430 (2018): 3802–3818. 29940187 10.1016/j.jmb.2018.06.033

[51] H. Chen , P. Yang , D. Yang , et al., “The PER3(rs772027021) SNP Induces Pigmentation Phenotypes of Dyschromatosis Universalis Hereditaria,” Journal of Molecular Medicine (Berlin, Germany) 101 (2023): 279–294. 36790533 10.1007/s00109-023-02288-6

[52] D. Zhou , P. Yang , and H. Chen , “Retyping and Molecular Pathology Diagnosis of Dyschromatosis Universalis Hereditaria,” Experimental Dermatology 32 (2023): 1334–1343. 37353900 10.1111/exd.14860

[53] M. Kono , K. Sugiura , M. Suganuma , et al., “Whole‐Exome Sequencing Identifies ADAM10 Mutations as a Cause of Reticulate Acropigmentation of Kitamura, a Clinical Entity Distinct From Dowling‐Degos Disease,” Human Molecular Genetics 22 (2013): 3524–3533. 23666529 10.1093/hmg/ddt207

[54] K. Okamura , Y. Abe , Y. Araki , Y. Hozumi , M. Kawaguchi , and T. Suzuki , “Behavior of Melanocytes and Keratinocytes in Reticulate Acropigmentation of Kitamura,” Pigment Cell & Melanoma Research 29 (2016): 243–246. 26708207 10.1111/pcmr.12453

[55] M. Kono and M. Akiyama , “Dyschromatosis Symmetrica Hereditaria and Reticulate Acropigmentation of Kitamura: An Update,” Journal of Dermatological Science 93 (2019): 75–81. 30692041 10.1016/j.jdermsci.2019.01.004

[56] C. Stephan , M. Kurban , and O. Abbas , “Dowling‐Degos Disease: A Review,” International Journal of Dermatology 60 (2021): 944–950. 33368260 10.1111/ijd.15385

[57] M. Kono , M. Suganuma , H. Takama , et al., “Dowling‐Degos Disease With Mutations in POFUT1 Is Clinicopathologically Distinct From Reticulate Acropigmentation of Kitamura,” British Journal of Dermatology 173 (2015): 584–586. 25639155 10.1111/bjd.13702

[58] D. J. Ralser , F. B. Basmanav , A. Tafazzoli , et al., “Mutations in γ‐Secretase Subunit‐Encoding PSENEN Underlie Dowling‐Degos Disease Associated With Acne Inversa,” Journal of Clinical Investigation 127 (2017): 1485–1490. 28287404 10.1172/JCI90667 PMC5373890

[59] M. Pavlovsky , O. Sarig , M. Eskin‐Schwartz , et al., “A Phenotype Combining Hidradenitis Suppurativa With Dowling‐Degos Disease Caused by a Founder Mutation in PSENEN,” British Journal of Dermatology 178 (2018): 502–508. 28922471 10.1111/bjd.16000

[60] S. Garcovich , P. M. Tricarico , C. Nait‐Meddour , et al., “Novel Nicastrin Mutation in Hidradenitis Suppurativa‐Dowling‐Degos Disease Clinical Phenotype: More Than Just Clinical Overlap?,” British Journal of Dermatology 183 (2020): 758–759. 32282940 10.1111/bjd.19121 PMC7586838

[61] H. Mehta , S. Gupta , M. Gupta , D. Chatterjee , and D. De , “Hidradenitis Suppurativa With Dowling‐Degos Disease Associated With KRT14 Mutation in an Indian Family,” Indian Dermatology Online Journal 16 (2025): 866–868. 40709850 10.4103/idoj.idoj_516_24 PMC12419722

[62] Z. J. Jaeger , N. Maverakis Ramirez , A. D. Osborne , et al., “RASopathies. Part I: Genetics and Therapeutic Considerations,” Journal of the American Academy of Dermatology, 2025, 10.1016/j.jaad.2025.05.1455. 40518121

[63] R. Halaban , “The Regulation of Normal Melanocyte Proliferation,” Pigment Cell Research 13 (2000): 4–14. 10761990 10.1034/j.1600-0749.2000.130103.x

[64] K. A. Rauen , “The RASopathies,” Annual Review of Genomics and Human Genetics 14 (2013): 355–369. 10.1146/annurev-genom-091212-153523 PMC4115674 23875798

[65] Z. J. Jaeger , N. K. A. Maverakis Ramirez , A. D. Osborne , et al., “RASopathies. Part II: Cutaneous and Extracutaneous Manifestations,” Journal of the American Academy of Dermatology, 2025, 10.1016/j.jaad.2025.01.106. 40532825

[66] D. Casey , S. Demko , A. Sinha , et al., “FDA Approval Summary: Selumetinib for Plexiform Neurofibroma,” Clinical Cancer Research 27 (2021): 4142–4146. 33712511 10.1158/1078-0432.CCR-20-5032

